# Possible transmission of HIV Infection due to human bite

**DOI:** 10.1186/1742-6405-8-16

**Published:** 2011-03-31

**Authors:** Alaka K Deshpande, Shivaji K Jadhav, Atmaram H Bandivdekar

**Affiliations:** 1 Department of Medicine and Head of ART Centre, Grant medical college and Sir J J Gr.of Govt Hospitals, Byculla, Mumbai - 400008 India; 2 Biochemistry Division, National Institute for Research in Reproductive Health (NIRRH), Indian Council of Medical Research (ICMR), J M Street, Parel, Mumbai - 400 012, India

## Abstract

The potential risk of HIV-1 infection following human bite although epidemiologically insignificant, but it is biologically possible. There are anecdotal reports of HIV transmission by human bites particularly if saliva is mixed with blood. The oral tissues support HIV replication and may serve as a previously unrecognized HIV reservoir. The HIV infected individuals have more viruses in blood than saliva, possibly due to the potent HIV-inhibitory properties of saliva. The case presented here is of a primary HIV infections following a human bite where in the saliva was not blood stained but it got smeared on a raw nail bed of a recipient. The blood and saliva of the source and blood of the recipient showed a detectable viral load with 91% sequence homology of C2-V3 region of HIV gp120 between the two individuals. The recipient did not receive PEP [post exposure prophylaxis] as his family physician was unaware of salivary transmission. The family physician should have taken PEP decision after proper evaluation of the severe and bleeding bite. Hence it is necessary to treat the HIV infected human bites with post exposure prophylaxis.

## Introduction

The epidemiological data has supported the premise that HIV transmission via saliva is low or non-existent due to inhibitory factors in saliva. The risk of HIV-1 infection following human bite although epidemiologically insignificant, but it is biologically possible [1,2]. Animal studies with rhesus macaques shows the infants are more susceptible to oral infections [3]. The oral trauma, co-infections with other sexually transmitted pathogens, periodontal diseases, concomitant ulcerative lesions, further enhance oral HIV transmission. The human bites as a rare risk factor for HIV transmission [4]. Some patients are hyper-excretors [5] they have high levels of infectious HIV in their saliva than in blood. These hyper-excretors may be at risk of transmitting the virus to their partners even though the blood viral load is low.

An interesting case of HIV transmission following a human bite is reported.

## Case report

On 29 March 2010, a 44 year's old man (Mr.A) a known case of NIDDM (Non-insulin dependent diabetes mellitus) and hypertension for past four years under treatment was brought to the hospital with history of high grade fever and increasing drowsiness for past four days.

Clinical examination revealed a drowsy febrile patient without focal neurological deficit or meningeal signs. A clinical diagnosis of malaria, metabolic encephalopathy with sepsis was made, later the patient had two episodes of generalized tonic-clonic convulsions and was treated with midazolam and Loarazepam followed by phosphenytoin along with IV Artesunate. Basic investigations were normal. Malarial parasites were not detected. Computerized tomography (CT) Brain showed nonspecific changes. CSF was suggestive of viral meningitis. Despite inj. Acyclovir drowsiness continued. He was negative for HBV, HCV, and HSV. The HIV Duo test was weakly positive. (Mr. A) did not provide any history of unprotected sex or multiple sex partners, nor any intravenous drug use in past or present, His spouse is HIV negative, The HIV-I viral load on the 4^th ^day of hospitalization was >750,000 copies/ml (Cobas Taqman 48 Real time PCR) and The CD4^+ ^cell count was 396 cells/mm^3^. A diagnosis of acute HIV infection was considered and TDF+FTC+EFV were started as per DHHS guidelines.

**Figure 1:**
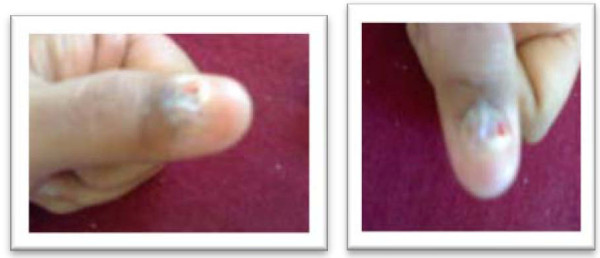
**shows the condition of the nail four weeks after the bite**.

As the sensorium improved, details from patient revealed that his foster son (Mr.X) who was HIV-1 positive, due to heterosexual acquisition. He was drug naïve with CD4 cell count 460 cells/mm^3. ^On 1^st ^March father and son duo, had an argument during which the son severely bit the left thumb of the father (Figure 1). During the incident the thumb nail of (Mr.A) came out leaving behind a raw bleeding nail bed.

Clinical examination of (Mr.X) revealed that his oral hygiene was good, absence of oral ulcers, caries no bleeding in gums. There were no physical injury, cuts or scratches occurred during the argument. The patient consulted his family physician who did not advice PEP, as salivary transmission of HIV is rare and negligible.

**Figure 2:**
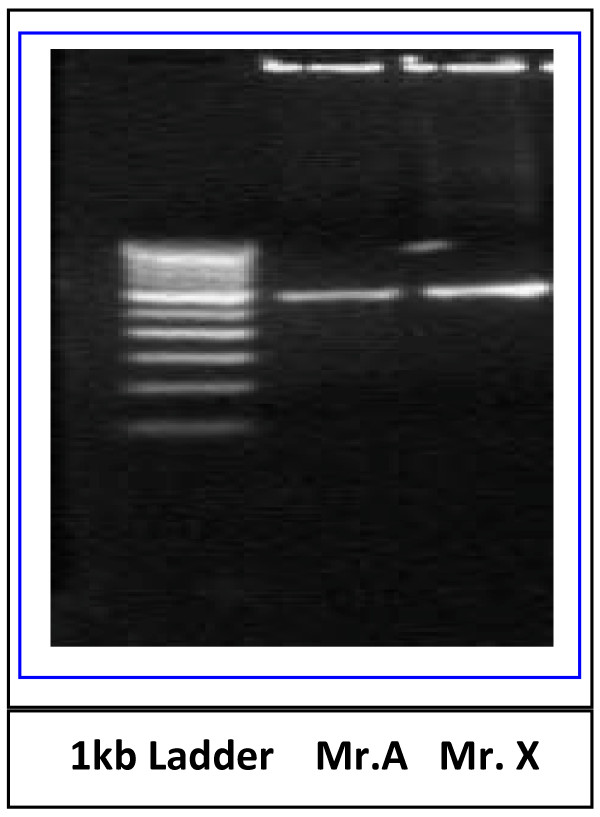
**PCR amplification of C2-V3 region of HIV1C *env *gene from PBMCs**.

On 29 March 2010, four weeks after the incidence.(Mr A) was hospitalized and his HIV Duo test was weakly positive and his viral load was high (2,470,000 copies/ml). The case thus indicates acquisition of HIV infection through saliva not contaminated with blood, following a human bite. The blood samples of both the father-son duo and saliva of the source (Mr.X) were collected on 12 April 2010, these samples were analyzed for confirmation of HIV DNA by amplifying C2-V3 Region of HIV1C envelope gene and further confirmed by PCR and sequencing. Peripheral blood mononuclear cells (PBMCs) were isolated by Ficoll-hypaque method. DNA from these PBMCs was extracted using Qiagen DNA mini kit and used for PCR [Qiagen, GmbH, Hilden, Germany]. The C2-V3 region of *env *gene was amplified (Figure 2) by nested PCR by heteroduplex mobility assay (HMA) using primers [6] obtained from NIH AIDS Research and Reference Reagent Programme (NIH, Bethesda, USA) to determine HIV variants and subtypes.

The C2-V3 region of second round product was sequenced. Sequence revealed 91% homology between Mr.A and Mr. X (Table 1). The C2-V3 region of HIV1C env gene shows the presence of five N-linked glycosylation (NLG) sites in (Mr A) While in case of (Mr.X) showed six potential NLG sites. The presence of single NLG site at V3 region of HIV 1C *env *gene showed in both the individuals suggesting the possible usages of CCR5 tropic virus.

**Table 1:** Translated Amino acid sequences of C2-V3 Region of HIV 1C *env *gene from Peripheral blood mononuclear cells (PBMCs)

ID	225	231	241	251	260
A	I**L**KCKD	**NTF NG**T	GPCN	*NVS*TVQCTHG	I**K**PVVSTQLL
X	I**I**KCND	**ETS N K**T	GPCN	*NVS*TVQCTHG	I**R**PVVSTQLL
					
	261	271	281	295	
A	L*NGS*LAE**EVV**	II-S**K***NLT*DN	**TE**TIIV**Q**L**D**E	**A**VEIT	
X	L*NGS*LAE**KEI**	II**R**S**E***NLT*DN	**AK**TIIV**H**L*N*E	**S **VEII	
					
	296	301	311	321	330
A	CTRPT	*NNT*RKSIRIG	PGQTFYATGD	IIGNIRRAYC	
X	CTRPN	*NNT*RKSIRIG	PGQTFYATGD	IIGNIRQAYC	

♦*italic *letters Indicate - NLG sites and **bold **letter indicates variation

♦Starts C2 region of HIV1gp120 followed by V3 region Starts at 296 and end at 330 positions

The virological and immunological parameters of Mr X and Mr A were measured (Table 2). The HIV-1 viral load in the blood and saliva was estimated using the MagNa pure Compact Nucleic Acid Automated System (Roche Diagnostic GmbH, Germany) with Cobas TaqMan 48 Real time PCR (Roche Molecular Systems Branchburg, NJ, USA).

**Table 2:** Virological and Immunological parameters

Sample ID	Sample	CD4 count Cells/μl	Viral Load Copies/ml
Mr.A (Pt.)	Blood plasma	396	2,470,000
	
Mr.X (Source)	Blood Plasma	383	17163
	
	Saliva	NA	2405
	
	Salivary Cells	NA	165

NA- not applicable

Evidence from the reports of Healthcare workers (HCWs) bitten by HIV infected toddlers highlights universal precautions should be taken [7]. The potential transmission of HIV 1 by human bite retroviruses has also been reported [8] Detection of HIV-1 in saliva has implications for case identification, clinical monitoring and surveillance for drug resistance [9] which also reveals detectable salivary HIV RNA may be a useful analyte for detection of HIV infection for monitoring responses to ARV therapy.

## Conclusions

Our observations revealed transmission of HIV infection from the smear of non-contaminated saliva of [Mr.X] on the raw and bleeding nail bed of (Mr.A) To conclude, the family physician should have taken PEP decision after proper evaluation of the severe and bleeding bite. Hence it is necessary to treat the HIV infected human bites with post exposure prophylaxis.

## Competing interests

The authors declare that they have no competing interests.

## Authors' contributions

AKD - Case identifications, clinical Aspects of the study, drafted the manuscript. SKJ - Experimental design, molecular biology experiments, DNA/RNA isolations, Viral load, PCR, Interpretation of data, Sequence analysis, AHB - Experimental design, data analysis, Interpretation of data, drafted manuscript

All the authors read and approved the final manuscript

## Consent

Written informed consent was obtained from the patient for publication of this case report and accompanying images. A copy of the written consent is available for review by the Editor-in-Chief of this journal.

## References

[B1] Tereskerz TM Bentley M Jagger J Risk of HIV-1 infection after human bites Lancet 1996 348 1512 10.1016/S0140-6736(05)65921-1 8942788

[B2] Richman KM Richman LS The potential for transmission of human immunodeficiency virus through human bites J Acquir Immune Defic Syndr 1993 64 40 46 8455145

[B3] Chenine AL Regina FF Lehmann H Vangel MG McClure HM Ruprecht RM Older Rhesus Macaque Infants Are More Susceptible to Oral Infection with Simian-Human Immunodeficiency Virus 89.6P than Neonates Virol 2005 2 1333 1336 10.1128/JVI.79.2.1333-1336.2005 PMC538536 15613361

[B4] Bartholomew CF Jones AM Human bites: a rare risk factor for HIV transmission AIDS 2006 4 631 632 10.1097/01.aids.0000210621.13825.75 16470132

[B5] Shugars DC Patton LL Freel SA Gray LR Vollmer RT Eron JJ Jr Fiscus SA Hyper-excretion of human immunodeficiency virus type 1RNA in saliva J Dent Res 2001 2 414 420 10.1177/00220345010800020301 11332524

[B6] Delwart EL Sheppard HW Walker BD Goudsmit J Mullins JI HIV-1 evolution *in vivo *tracked by DNA heteroduplex mobility assays J Virology 1994 68 6672 6683 8084001 10.1128/jvi.68.10.6672-6683.1994 PMC237088

[B7] Shirly LR Ross SA Risk of transmission of human immunodeficiency virus by bite of an infected toddler J Pediatrics 1989 3 424 427 10.1016/s0022-3476(89)80563-3 2921686

[B8] Andreo SM Barra LA Costa LJ Sucupira MC Souza E Diaz RC HIV type 1 transmission by human bite retroviruses AIDS Res Hum Retroviruses 2004 20 349 350 10.1089/088922204323048087 15157352

[B9] Balamane M Winters MA Dalai SC Freeman AH Traves MW Israelski DM Katzenstein DA Klausner JD Detection of HIV-1 in saliva: Implications for case-Identification, clinical monitoring and surveillance for drug resistance The open Virology Journal 2010 4 78 83 10.2174/1874357901004010088 PMC3111737 21673840

